# Epistemonikos: a comprehensive database of systematic reviews for health decision-making

**DOI:** 10.1186/s12874-020-01157-x

**Published:** 2020-11-30

**Authors:** Gabriel Rada, Daniel Pérez, Felipe Araya-Quintanilla, Camila Ávila, Gonzalo Bravo-Soto, Rocío Bravo-Jeria, Aldo Cánepa, Daniel Capurro, Victoria Castro-Gutiérrez, Valeria Contreras, Javiera Edwards, Jorge Faúndez, Damián Garrido, Magdalena Jiménez, Valentina Llovet, Diego Lobos, Francisco Madrid, Macarena Morel-Marambio, Antonia Mendoza, Ignacio Neumann, Luis Ortiz-Muñoz, José Peña, Marcelo Pérez, Franco Pesce, Carmen Rain, Solange Rivera, Javiera Sepúlveda, Mauricio Soto, Felipe Valverde, Juan Vásquez, Francisca Verdugo-Paiva, Camilo Vergara, Cynthia Zavala, Ricardo Zilleruelo-Ramos, on behalf of Epistemonikos project

**Affiliations:** 1 Centro Evidencia UC, Pontificia Universidad Católica de Chile, Santiago, Chile; 2 Epistemonikos Foundation, Avenida Holanda 895, Providencia, Santiago, Chile; 3 Rehabilitation in Health Research Center (CIRES), Universidad de las Américas, Santiago, Chile; 4 Faculty of Health Sciences, Universidad SEK, Santiago, Chile; 5 School of Computing and Information Systems, University of Melbourne, Melbourne, Australia; 6 Department of Clinical Epidemiology and Biostatistics, McMaster University, Hamilton, Canada; 7 Living Knowledge SpA, Santiago, Chile

**Keywords:** Bibliographic database, Systematic reviews, Epistemonikos, Evidence-based practice

## Abstract

**Background:**

Systematic reviews allow health decisions to be informed by the best available research evidence. However, their number is proliferating quickly, and many skills are required to identify all the relevant reviews for a specific question.

**Methods and findings:**

We screen 10 bibliographic databases on a daily or weekly basis, to identify systematic reviews relevant for health decision-making. Using a machine-based approach developed for this project we select reviews, which are then validated by a network of more than 1000 collaborators. After screening over 1,400,000 records we have identified more than 300,000 systematic reviews, which are now stored in a single place and accessible through an easy-to-use search engine. This makes Epistemonikos the largest database of its kind.

**Conclusions:**

Using a systematic approach, recruiting a broad network of collaborators and implementing automated methods, we developed a one-stop shop for systematic reviews relevant for health decision making.

**Supplementary Information:**

The online version contains supplementary material available at 10.1186/s12874-020-01157-x.

## Summary points

- A landmark study in 2010 estimated that 11 systematic reviews were published each day. Other researchers have reported an exponential growth of epidemic proportions afterwards. Our estimate is that 104 systematic reviews relevant for health decision-making are currently published each day.
- The growth of systematic reviews and the skills needed to retrieve them from across multiple databases make it almost impossible for the scientific community, health care providers and policymakers to keep up.
- Using a systematic approach, which includes a broad network of collaborators and the use of automated methods, we developed Epistemonikos, an easy-to-use, one-stop shop for systematic reviews relevant for health decision-making.

## Background

A wide consensus has been reached about the need for making health decisions informed by the best available research evidence. Such decisions help to assure quality and efficiency and maximise the benefits while minimising harms and costs [1].

For most health questions there is already a substantial number of studies that can inform decisions. In fact, in several areas the information exceeds what clinicians or policymakers trying to keep up with the evidence can handle [2]. Systematic reviews and meta-analyses (henceforth referred to as systematic reviews) were invented to deal with the problem of having several studies answering a similar question. A well-conducted systematic review is considered the best available evidence according to Evidence-Based Health Care methodology [3].

Because systematic reviews are highly valued by the scientific community and health care providers, their number has increased. Other incentives, such as systematic reviews being relatively easy to perform and publish in high impact journals [4] and becoming a marketing tool [5] have also fueled their proliferation. A landmark study estimated their production at 11 per day in 2010 [2], and others have reported an exponential growth afterwards [4].

However, clinicians and other decision-makers lack the skills to search in biomedical databases [6, 7], and there is not a single source that provides all the relevant systematic reviews [8].

An easy-to-use, comprehensive database for systematic reviews would allow the scientific community, health care providers and policymakers to find and use the best available research evidence efficiently in the limited time they have to make a decision. The Database of Abstracts of Reviews of Effects (DARE), maintained by the Centre for Reviews and Dissemination, University of York, UK, intended to play this role, but it was discontinued in March 2015 [9].

Epistemonikos is a collaborative project started in 2009 with the objective of gathering, organising and comparing all of the relevant research evidence for health decision-making in a single database [10]. This article describes the methods designed and the results obtained during the first stage of the project which consists in identifying all of the relevant systematic reviews.

## Construction and content

We built our methods by following or adapting the Preferred Reporting Items for Systematic Reviews and Meta-Analyses (PRISMA) [11] and the Cochrane Handbook [12].

### Criteria for considering systematic reviews for Epistemonikos Database

In accordance with the Cochrane Collaboration and the PRISMA Statement [11, 12], we have adopted the following definition: ‘A systematic review attempts to collate all empirical evidence that fits pre-specified eligibility criteria to answer a specific research question. It uses explicit systematic methods that are selected with a view to minimising bias, thus providing reliable findings from which conclusions can be drawn and decisions made’ [12].

The operational criteria to consider a systematic review for inclusion in Epistemonikos Database are:

1. Its main purpose is to synthesise primary studies.
2. It states at least one explicit eligibility criterion.
3. It reports searching in at least one electronic database.

Additionally, we include any synthesis of primary studies that do not fulfil the above definition but is judged to add valuable information, such as individual patient or unpublished data meta-analysis where studies have not been identified through a systematic search process.

Evidence-syntheses that fulfil criteria 3 but not all of the above are not excluded from Epistemonikos Database but are classified under a different category (i.e. broad synthesis plus a specific subtype, such as guideline, overview of systematic reviews), which is not the subject of this article.

We exclude reviews that:

- Do not address a human health problem.
- Synthesise studies that do not evaluate individuals or groups of individuals (e.g. preclinical or animal studies).
- Explore a methodological issue (i.e. research about research).
- Are only presented as conference abstracts.

### Search methods for identification of systematic reviews

#### Electronic databases

Epistemonikos was developed and is maintained by systematically searching 10 databases in a daily or weekly basis: Cochrane Database of Systematic Reviews, Pubmed/MEDLINE, EMBASE, CINAHL, PsycINFO, LILACS, DARE, Campbell library, JBI Database of Systematic Reviews and Implementation Reports and EPPI-Centre Evidence Library.

We do not restrict our search by language, publication status or publication date (i.e. databases have been searched from inception). In the case of databases of structured summaries (i.e. DARE database), we retrieve the article being summarised and assess it using the same inclusion criteria.

The search strategies were pragmatically adapted from previously reported strategies to retrieve systematic reviews [13] and improved by a team of search experts who analysed the search terms obtained from the text mining of relevant and irrelevant records.

The detailed search strategies currently used in Epistemonikos Database are described in additional file 1.

#### Other sources

In order to identify systematic reviews potentially missed by our search in electronic databases we:

1. Include systematic reviews identified in overviews of reviews, guidelines, scoping reviews or other types of broad syntheses (which are also included in Epistemonikos Database but are classified under a different category).
2. Check references of selected included reviews.
3. Run cross-citation searches in Google Scholar and Microsoft Academic.
4. Evaluate potentially eligible reviews sent by users through the contact page or other means (e.g. email, twitter).

### Data collection and analysis

#### Selection of reviews

The selection is conducted in two steps. First, all potentially eligible articles are classified as they enter the database using automated methods specifically created for this project (a machine learning classifier for the records with an abstract and a heuristic classifier for the records without an abstract). Secondly, a collaborative network of Epistemonikos users validates this classification. Records with a high probability of being false positives or false negatives are regularly checked by a dedicated team of method experts.

#### Development of the classifier for records with an abstract

The dataset used to develop the classifier includes all the records with an abstract that had been manually screened by at least one reviewer by January 2019. This dataset was formed by 102,011 systematic reviews and 42,321 records not corresponding to systematic reviews, most of them classified before 2016 when Epistemonikos Database selection process was conducted only by human screeners (earlier versions of the classifier had been in use during 2017 and 2018).

The dataset was arbitrarily divided into two splits as training and validation (80 and 20% respectively). The training split was used to build a classifier using a supervised learning random forest and the validation split was used to test its predictive power [14]. The terms composing the classifier were iteratively analysed and improved by a team of software engineers with expertise in information retrieval, methodologists and information specialists until reaching a stable version. Finally, the results were manually validated with a set of 500 unseen records to make sure we had not overfit the model during the tuning of the random forest model.

#### Development of the classifier for records without an abstract

Acknowledging the limitations of any classifier using a language-based technique to manage records without an abstract, we approached these as a separate problem. We reviewed the sample iteratively to identify characteristics associated with a high probability of being or not being a systematic review and custom-built a heuristic classifier using specific terms and other characteristics of the records.

#### Automated classification (classifiers)

All the records retrieved by the search strategy are immediately processed and automatically classified into included/excluded systematic reviews in the database. Later, the classifications are manually validated by at least one human screener.

#### Human validation

All the titles and abstracts included by the classifier are uploaded to Collaboratron™ [15], a screening software specifically developed by Epistemonikos Foundation for this purpose. The documents are screened by at least one human using this tool, starting from the most recent records. The records without an abstract are regularly reviewed by a dedicated team. The full text of the article is retrieved if it is not possible to make a decision based on the title or abstract.

Discrepancies between the classifier and a human screener (i.e. included by the classifier and excluded by the human screener) or between different human screeners are resolved by a senior researcher.

### Measures of performance

In order to estimate the performance of the classifiers, we used the validation set as a gold standard for the machine learning classifier and a convenience sample of 500 unseen records for the heuristic classifier. We calculated the following measures (and their 95% confidence interval): sensitivity or recall (true positives/(true positives + false negatives)), precision or positive predictive value (true positives/(true positives + false positives)), specificity (true negatives/(false positives + true negatives)) and accuracy ((true positives + true negatives)/total). For estimation of misclassified reviews in Epistemonikos Database, we applied these numbers to the total amount of records without human validation.

## Utility and discussion

### Results of the search

On January 24, 2020, the literature search had retrieved 1,431,972 records, which after removing duplicates correspond to 704,150 potentially eligible systematic reviews (626,121 with and 78,029 without an abstract). The total number of included systematic reviews in Epistemonikos is 307,119 (104,050 already validated by at least one human). We have identified 13,369 reviews from sources different than the search strategy in electronic databases.

**Fig. 1:**
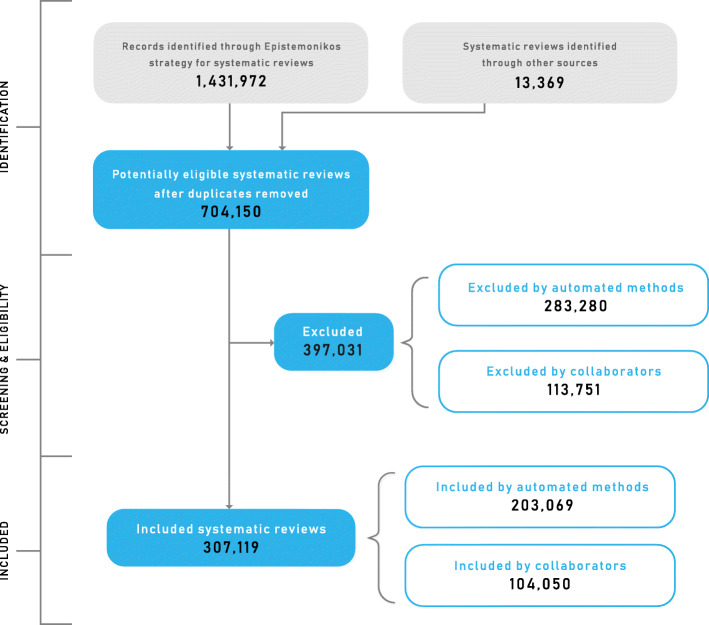
PRISMA flow diagram

A flow diagram summarizing the screening and selection process is presented in Fig. 1**.** A living report (updated daily) can be consulted at https://www.epistemonikos.org/about_us/updated_report

The number of systematic reviews has increased at a rate of 18.2% per year since 1990. In the last decade (January 1st, 2010 to December 31st, 2019), it went from 11,233 per year to 37,944, that is a 3.5-fold increase. On average, 104 systematic reviews are published each day.

**Fig. 2:**
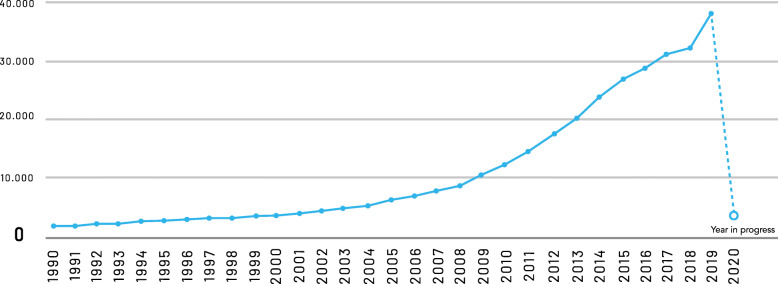
Number of systematic reviews per year

The number of systematic reviews per year is shown in Fig. 2.

### Performance of the classifiers

The sensitivity of the machine learning classifier for the detection of systematic reviews in records with an abstract was 96.8% (95% CI, 96.58 to 97.06%), the specificity was 80.4% (95% CI, 79.55 to 81.23%), and the accuracy was 92.0% (95% CI, 91.65 to 92.28%). The sensitivity of the heuristic classifier for the detection of systematic reviews in records without an abstract was 94.1% (95% CI, 88.74 to 96.97%); the specificity was 55.6 (95% CI, 50.49 to 60.63%); and the accuracy was 66.0% (95% CI, 61.74 to 70.02%).

Applying these figures to the records that have only been evaluated by the classifiers, the number of actual systematic reviews missing in Epistemonikos Database (false negatives) can be estimated at 9122 (3.0%), and the number of records classified erroneously as systematic reviews (false positives) can be estimated at 44,242 (14.4%).

The complete performance of the classifiers is presented in Table 1.

**Table 1:** Classification accuracy of the automated approach

Measure	Value (95% CI)
Machine learning classifier (for records with abstract)	
Sensitivity	96.8% (96.58 to 97.06%)
Specificity	80.4% (79.55 to 81.23%)
Precision	92.2% (91.79 to 92.51%)
Accuracy	92.0% (91.65 to 92.28%)
Heuristic classifier (for records without abstract)	
Sensitivity	94.1% (88.74 to 96.97%)
Specificity	55.6% (50.49 to 60.63%)
Precision	43.9% (38.34 to 49.71%)
Accuracy	66.0% (61.74 to 70.02%)

## Conclusions

### Estimation of the number of systematic reviews

Our project confirms the dramatic increase in the number of systematic reviews that has been reported by other researchers [2, 4, 5]. A well-known study [2] using data from 2007 estimated the average number of published systematic reviews at 11 per day. Our estimation is slightly superior during the same period (17.6 reviews per day), which might be partially explained by the rate of false positives in Epistemonikos or by an underestimation of the numbers in the former. Moreover, the difference might reflect the larger number of search sources and improved strategies developed by Epistemonikos. More recent studies have reached to lower [16] or higher [5] estimations, probably because of the variation in the methods employed. For instance, some authors have screened a sample of potentially eligible reviews in PubMed (i.e. records published during a one-month period) [16] or simply counted PubMed records tagged as systematic review or meta-analysis [5]. The use of different definitions of what is considered a systematic review might also explain the differences.

### Comparison against other databases/repositories of systematic reviews

In comparison with other searchable and freely available repositories of systematic reviews, Epistemonikos Database has multiple advantages. In terms of comprehensiveness, it contains substantially more systematic reviews than any other repository. For instance, DARE [9] included about 45,000 reviews when it was discontinued in 2015, TripDatabase contains about 65,000 reviews [17], and there were over 40,000 systematic reviews in PubMed Health when it was discontinued in 2018 [18]. In 2019 PubMed announced the addition of a new publication type MeSH term for systematic reviews [19], which retrieves about 110,000 citations as of January 2020.

Another advantage of our approach is the transparency in the reporting of methods and results. A systematic approach and a clear report make it possible to be certain on the contents of the database and to be aware of its limitations. In terms of reporting, we have adapted standards for systematic reviews [11], and we have developed the technology to apply those standards to a massive amount of records. Our PRISMA flowchart, for example, is updated daily, even though it is one of the largest, if not the largest project to be summarised in this format.

### One-stop shop

Probably the more important question, for both researchers and decision-makers, is if they can rely on Epistemonikos Database and not conduct their own searches elsewhere. One study comparing searches for systematic reviews in 7 key databases, including Epistemonikos, concluded that no single database was able to retrieve all of the reviews used as a reference set [8]. Epistemonikos was selected as part of a combination of 3 databases which, combined, retrieved the highest number of unique records. However, it is important to note that this comparison was made when Epistemonikos was at an early stage, before the current methods and technologies were fully deployed, and included only 60,000 reviews (currently, there are 198,000 records in the database that would have been available at the time of that comparison). So, if a similar study were conducted nowadays, it is reasonable to expect a substantially better recall.

### Limitations of this article

The main limitation of this article is that the performance of Epistemonikos has not been estimated using a proper gold standard, such as the one used in previous studies [8, 13]. Recent studies by our research group are addressing this limitation and have preliminary shown Epistemonikos includes most of the reviews that would be retrieved when using an exhaustive approach as gold standard [20, 21]. We think it is reasonable to affirm Epistemonikos constitutes a one-stop shop for systematic reviews from the perspective of most users but more studies are needed in order to establish if it can be used as a unique source for reviews in more rigorous contexts, such as the conduction of guidelines or overviews of systematic reviews.

### Limitations of Epistemonikos Database

The proliferation of systematic reviews has been described as one of epidemic proportions [5] and as a mega-silliness [22], and portrayed as a massive production of unnecessary, misleading, and conflicted evidence [5, 22]. Nevertheless, systematic reviews are recognised as an invaluable tool to make health decisions [11], as a key source of information in policymaking [23], as the staple of practice guidelines [24], as the main input for textbooks and point-of-care tools supporting health professionals and students [25], and as the most effective way to prioritise research needs and to reduce waste in evidence production [26].

In this context, a one-stop shop of systematic reviews is a *conditio* *sine* *qua* *non* for the adoption of an evidence-based decision-making model in real-life practice and policy making. However, most of the existing systematic reviews have major limitations in terms of currency and quality [5, 23, 27, 28], so still, a substantial effort by users to separate the wheat from the chaff is required. This problem is not yet addressed by Epistemonikos Database.

In the current scenario, users will often need to critically appraise dozens of overlapping reviews to answer a single question [29], most of them of poor quality [5] or out of date [27, 28]. This represents an insurmountable barrier to the adoption of evidence-based decision-making.

Furthermore, even with this massive production of systematic reviews there is a large proportion of primary studies that are not yet covered by systematic reviews, which are not routinely collected by Epistemonikos [30]. The lack of coordination in the review production has led researchers to cover much the same territory.

The present state of a wide acceptance of systematic reviews amidst important challenges to their development has been portrayed as a ‘midlife crisis’ [31]. The transition to a mature field requires that we expand the existing efforts to improve the quality and reporting of reviews [11], establish initiatives to discourage the conduct of redundant reviews [32], and promote the adoption of better and more sustainable review models [33].

## Supplementary Information

**Additional file 1.**

## Data Availability

The datasets used and analysed during the current study or datasets needed to reproduce the results of this study (e.g. a list of included/excluded records) are available from the corresponding author on reasonable request.
