# Supplementary material for: Epistemonikos: a comprehensive database of systematic reviews for health decision-making
Source: BMC Med Res Methodol. 2020 Nov 30;20:286. doi: 10.1186/s12874-020-01157-x (PMC7708132; doi:10.1186/s12874-020-01157-x)
Supplement: Supplementary file 1 — Additional file 1. [file 12874_2020_1157_MOESM1_ESM.pdf]

Additional file 1 - Search strategies

Cochrane Library - Cochrane database of systematic reviews (CDSR)

http://www.thecochranelibrary.com

Frequency of search: daily

Search strategy:

#1 * , in Title, Abstract, Keywords: Cochrane Reviews (Reviews NOT protocols)

Medline/PubMed - US National Library of Medicine

http://www.ncbi.nlm.nih.gov/pubmed/

Frequency of search: daily

Search strategy:

#1 ("critical review"[ti] OR "electronic search" OR "evidence-based analysis"[ti] OR "evidence-based review"[ti] OR "literature search"[ti] OR "meta analysis"[ti] OR "meta synthesis"[ti] OR "meta-analyse" OR "meta-analytic review"[ti] OR "meta-study"[ti] OR "meta-synthesis"[ti] OR "metaanalysis"[ti] OR "metasynthesis"[ti] OR "pooled effect"[ti] OR "random-effects model" OR "systematic quantitative review"[ti] OR "systematically searched" OR "systemic review" OR (review[ti] AND randomized[ti]) OR (systematic[Title/Abstract] AND review[Title/Abstract]) OR MEDLINE[Title/Abstract] OR meta analysis[pt] OR systematic review[pt] OR systematic[sb] OR "meta-analysis"[Title/Abstract] OR "literature review"[Title/Abstract] OR PubMed[Title/Abstract])

EMBASE (Excerpta Medica dataBASE)

Host: OVID

Frequency of search: weekly

Search strategy:

#1 "critical review".ti. or "electronic search".mp. or "evidence-based analysis".ti. or "evidence-based review".ti. or "literature search".ti. or "meta analysis".ti. or "meta synthesis".ti. or "meta-analyse".mp. or "meta-analytic review".ti. or "meta-study".ti. or "meta-synthesis".ti. or "metaanalysis".ti. or "metasynthesis".ti. or "pooled effect".ti. or "random-effects model".mp. or "systematic quantitative review".ti. or "systematically searched".mp. or "systemic review".mp. or (review and randomized).ti. or (systematic and review).ti,ab. or MEDLINE.ti,ab. or meta analysis.pt. or systematic review.pt. or "meta-analysis".ti,ab. or "literature review".ti,ab. or PubMed.ti,ab.

CINAHL (Cumulative Index to Nursing and Allied Health Literature)

Host: EBSCO

Frequency of search: weekly

Search strategy:

#1 TI "critical review" OR electronic search OR TI "evidence-based analysis" OR TI "evidence-based review" OR TI "literature search" OR TI "meta analysis" OR TI "meta synthesis" OR meta-analyse OR TI "meta-analytic review" OR TI "meta-study" OR TI "meta-synthesis" OR TI "metaanalysis" OR TI "metasynthesis" OR TI "pooled effect" OR random-effects model OR TI "systematic quantitative review" OR systematically searched OR systemic review OR (TI review AND TI randomized) OR ((TI systematic OR AB systematic) AND (TI review OR AB review)) OR (TI MEDLINE OR AB MEDLINE) OR PT "meta analysis" OR PT "systematic review" OR (TI "meta-analysis" OR AB "meta-analysis") OR (TI "literature review" OR AB "literature review") OR (TI PubMed OR AB PubMed)

PsycINFO

Host: EBSCO

Frequency of search: weekly

Search strategy:

#1 TI "critical review" OR electronic search OR TI "evidence-based analysis" OR TI "evidence-based review" OR TI "literature search" OR TI "meta analysis" OR TI "meta synthesis" OR meta-analyse OR TI "meta-analytic review" OR TI "meta-study" OR TI "meta-synthesis" OR TI "metaanalysis" OR TI "metasynthesis" OR TI "pooled effect" OR random-effects model OR TI "systematic quantitative review" OR systematically searched OR systemic review OR (TI review AND TI randomized) OR ((TI systematic OR AB systematic) AND (TI review OR AB review)) OR (TI MEDLINE OR AB MEDLINE) OR PT "meta analysis" OR PT "systematic review" OR (TI "meta-analysis" OR AB "meta-analysis") OR (TI "literature review" OR AB "literature review") OR (TI PubMed OR AB PubMed)

LILACS (Literatura Latinoamericana y del Caribe en Ciencias de la Salud)

Host: BVS (http://lilacs.bvsalud.org/en/)

Frequency of search: weekly

Search strategy (1):

#1 (ti:(critical review)) OR (ti:(revisão crítica)) OR (ti:(revisión crítica)) OR (tw:(electronic search)) OR (tw:(busca eletrônica)) OR (tw:(busqueda electronica)) OR (ti:(evidence-based analysis)) OR (ti:(análise baseada em evidências)) OR (ti:(análisis basado en la evidencia)) OR (ti:(evidence-based review)) OR (ti:(revisão baseada em evidências)) OR (ti:(revisión basada en la evidencia)) OR (ti:(literature search)) OR (ti:(busca literaria)) OR (ti:(busqueda de literatura)) OR (ti:(meta analysis)) OR (ti:(meta-análise)) OR (ti:(metaanálisis)) OR (ti:(meta synthesis)) OR (ti:(meta síntese)) OR (ti:(meta síntesis)) OR (tw:(meta-analyse)) OR (tw:(meta-análise)) OR (tw:(metaanálisis)) OR (ti:(meta-analytic review)) OR (ti:(revisão meta-analítica)) OR (ti:(revisión meta-analítica)) OR (ti:(meta-study)) OR (ti:(meta-estudo)) OR (ti:(meta-estudio)) OR (ti:(meta-synthesis)) OR (ti:(meta-síntese)) OR (ti:(meta-síntesis)) OR (ti:(metaanalysis)) OR (ti:(metanálises)) OR (ti:(metaanálisis)) OR (ti:(metasynthesis)) OR (ti:(metassíntese)) OR (ti:(metasíntesis)) OR (ti:(pooled effect)) OR (ti:(efeito combinado)) OR (ti:(efecto combinado)) OR (tw:(random-effects model)) OR (tw:(modelo de efeitos aleatórios)) OR (tw:(modelo de efectos aleatorios)) OR (ti:(systematic quantitative review)) OR (ti:(revisão quantitativa sistemática)) OR (ti:(revisión cuantitativa sistemática)) OR

(tw:(systematically searched)) OR (tw:(sistematicamente pesquisado)) OR (tw:(buscar sistemáticamente)) OR (tw:(systemic review)) OR (tw:(revisão sistemática)) OR (tw:(revisión sistematica)) OR (ti:(review AND randomized)) OR (ti:(revisão e randomização)) OR (ti:(revisión y aleatorizado)) OR ((tw:(systematic)) AND (tw:(review))) OR ((tw:(sistemático)) AND (tw:(revisão))) OR ((tw:(revisión)) AND (tw:(sistemática))) OR (tw:(MEDLINE)) OR (pt: (meta analysis)) OR (pt: (meta análise)) OR (pt:(meta análisis)) OR (pt:("systematic review")) OR (pt: ("revisão sistemática")) OR (pt: ("revisión sistemática")) OR (tw:(meta-analysis)) OR (tw:(meta-análise)) OR (tw:(metaanálisis)) OR (tw:(literature review)) OR (tw:(revisão da literatura)) OR (tw:(revision de literatura)) OR (tw:(pubmed))

DARE (Database of Abstracts of Reviews of Effectiveness) - Centre for Reviews and Dissemination, University of York

Host: CRD (http://www.crd.york.ac.uk/CRDWeb/)

Search date: 2015 (no new records have been added to DARE after 2015)

#1 * , in Any field: CRD assessed review (bibliographic)/ CRD assessed review (full abstract)

The Campbell Collaboration Online Library

Host: Campbell Library (https://www.campbellcollaboration.org/library.html)

Frequency of search: daily

Search strategy:

#1 * , in Title: Review

JBI Database of Systematic Reviews and Implementation Reports

Searched through Pubmed (see above)

Frequency of search: daily

Search strategy:

#1 ("JBI Database System Rev Implement Rep"[jour] OR "JBI Libr Syst Rev"[jour]) NOT (("meta-analysis protocol"[ti] OR "review protocol"[ti] OR "scoping review protocol"[ti] OR "protocol for a systematic review"[ti] OR "evidence protocol"[ti] OR "implementation project"[ti]) OR (protocol AND (systematic OR meta-analysis OR umbrella OR scoping) AND (review OR evidence)))

EPPI-Centre Evidence Library

http://eppi.ioe.ac.uk/cms/Default.aspx?tabid=56

Frequency of search: weekly

Search strategy:

All records in chronological list (http://eppi.ioe.ac.uk/cms/Default.aspx?tabid=62)
